# Walking to Work: Trends in the United States, 2005–2014

**DOI:** 10.5888/pcd13.160181

**Published:** 2016-09-22

**Authors:** Yong Yang

## Abstract

I examined trends from 2005 through 2014 in walking to work compared with other modes of travel. For each year, I calculated the percentage of travel to work by private vehicle, public transportation, and walking and used distance decay functions to analyze the distribution of walking by distance. I found that the percentage of travel to work by walking remained stable, with a slight increase over time, and that people tended to walk longer to get to work. The trend is positive and encouraging, although more evidence is needed to confirm my findings.

## Objective

In the United States, the percentage of trips to work that are accomplished by walking has decreased over the past half century (1). Although National Household Travel Survey (2) data showed the percentage of walking trips of all types increased slightly from 2001 through 2009 (3), the overall trend in the past decade is not clear. The objective of this study was to examine the trend of walking trips to work from 2005 through 2014 by using data from the American Community Survey (ACS) (4). For each year, distance decay functions were used to analyze the distribution of walking by distance.

## Methods

I obtained the percentage of trips to work by walking from the ACS for each year from 2005 through 2014. ACS is an ongoing nationwide survey conducted by the US Census Bureau, which was fully implemented in 2005 and surveys approximately 3 million households per year. People were asked how they typically traveled to work during the previous week and their transportation mode, departure time, trip duration, and workplace location. For comparison purposes, I also obtained from the US Census the percentage of travel to work by walking for each decade from 1960 through 2000.

To describe mathematically the distribution of the length of walking trips, I used distance decay functions, which have been applied frequently in active travel (5–8). The main benefit of using distance decay functions is that they can be used to compare the distribution of walking distances among groups or the changes over time better than can the variables of mean or median. A negative exponential form was chosen (5,9) because the shorter the length, the more likely people are to select walking as a travel mode and because most walking trips occur within very short distances compared with other travel modes. I used walking duration as the proxy for trip length, because the latter is not available in ACS. The distance decay function is specified as:

*P* (*d*) = *e^−^*
^β^
*^d^*

Where *P* (*d*) denotes the cumulative percentage of walking trips with duration equal to or longer than the value of *d* (in minutes), and β is the decay parameter to be estimated using empirical data. For a specific duration *d*, smaller β leads to larger *P*, which indicates a larger percentage of walking trips with duration equal or longer than *d*.

## Results

**Figure 1:**
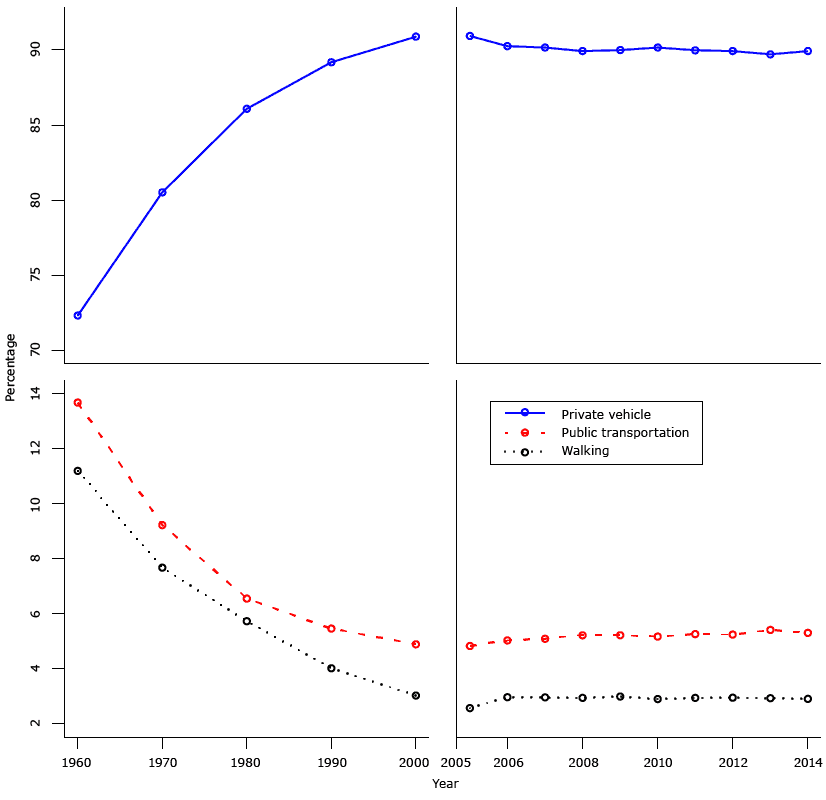
Percentage of travel to work by private vehicle, public transportation, and walking from 1960 through 2000, by decade, and from 2005 through 2014, by year, American Community Survey, United States.

Figure 1 shows the percentages of travel to work using private vehicle, public transportation, and walking for 2 periods, from 1960 through 2000 with data at each decade, and from 2005 to 2014 with data at each year. These 2 periods show totally different patterns. The percentage of travel to work by private vehicle increased steadily from approximately 72% in 1960 to more than 90% in 2000, while at the same time, the percentage of travel to work by public transportation and by walking decreased abruptly, from more than 13% in 1960 to approximately 5% in 2000 for public transportation and from 11% in 1960 to 3% in 2000 for walking. However, during the period from 2005 through 2014, the percentage of 3 travel modes remained relatively stable, with a slight decrease in private vehicle travel and a slight increase in walking.

**Table:** 

Year	Private Vehicle	Public Transportation	Walking
**1960**	**72.32**	**13.67**	**11.19**
**1970**	**80.52**	**9.22**	**7.67**
**1980**	**86.08**	**6.55**	**5.73**
**1990**	**89.18**	**5.46**	**4.02**
**2000**	**90.88**	**4.89**	**3.03**
**2005**	**90.93**	**4.83**	**2.57**
**2006**	**90.25**	**5.03**	**2.97**
**2007**	**90.16**	**5.09**	**2.96**
**2008**	**89.92**	**5.22**	**2.94**
**2009**	**89.99**	**5.22**	**2.99**
**2010**	**90.16**	**5.17**	**2.9**
**2011**	**89.98**	**5.26**	**2.94**
**2012**	**89.92**	**5.24**	**2.95**
**2013**	**89.71**	**5.41**	**2.93**
**2014**	**89.92**	**5.3**	**2.91**

**Figure 2:**
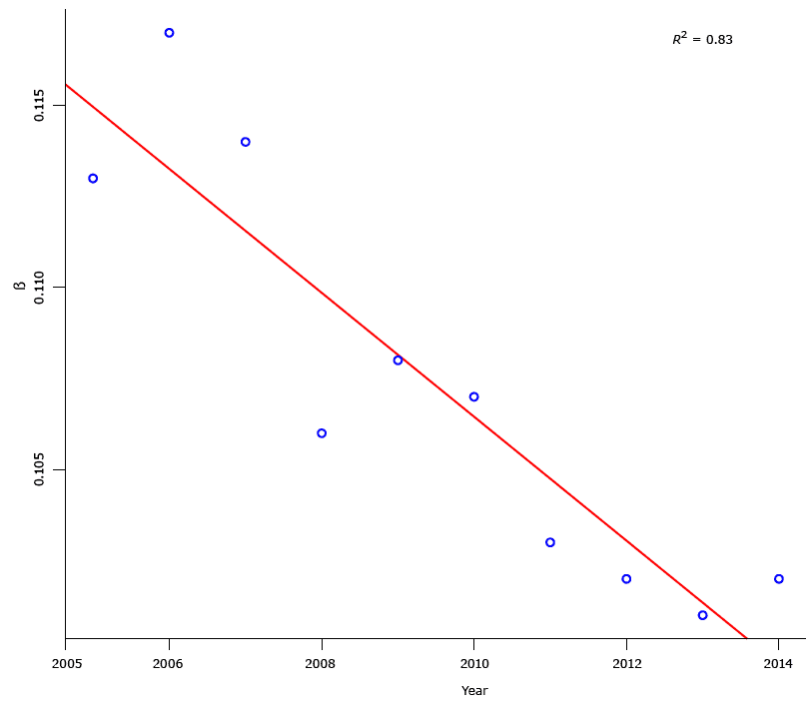
The β values of decay functions for the distribution of walking trips to work, by duration, American Community Survey, United States, 2005–2014. The figure also includes a trend line to show the change pattern over years. The trend line is negative, that is, it shows a decreasing pattern for the β value of decay functions over years. The *R* ^2^ value of 0.83 is the fitness of the trend line for the β values from 2005 through 2014. **Table:** 

In general, the duration of walks to work tended to increase over time from 2005 through 2014 (Table). For example, walking trips to work of no less than 10 minutes’ duration increased from 48% in 2005 to 54% in 2014. Decay parameters and the corresponding decay parameters for each year (with a negative fitted linear trend line, and *R* ^2^ value of 0.83) (Figure 2) provide an indicator of the overall length distribution pattern. This decreased tendency of decay parameter β indicates that the time walked to work increased over time.

**Table:** Distribution of US Walking Trips to Work by Duration, by the Fitted Distance Decay Function, American Community Survey, United States, 2005–2014^a^

Measurement		Percentage by Year									
		2005	2006	2007	2008	2009	2010	2011	2012	2013	2014
Minutes walked	<10	52.1	52.8	51.8	49.2	49.9	49.3	47.9	47.4	47.4	46.4
	10–14	70.2	71.6	71.0	68.7	69.4	68.9	67.8	67.4	67.2	66.2
	15–19	82.9	83.8	83.2	81.8	82.0	81.9	81.1	80.9	80.3	79.8
	20–24	90.7	91.0	90.4	89.7	89.5	89.7	88.9	88.7	88.4	88.0
	25–29	92.5	92.9	92.4	91.6	91.5	91.7	90.9	90.9	90.6	90.3
	30–34	97.0	97.0	96.8	96.4	96.3	96.4	95.9	95.9	95.6	95.6
	35–44	97.9	98.0	97.9	97.5	97.4	97.6	97.1	97.2	97.0	97.0
	45–59	99.0	99.0	98.9	98.6	98.6	98.8	98.4	98.5	98.4	98.4
	≥60	100.0	100.0	100.0	100.0	100.0	100.0	100.0	100.0	100.0	100.0
Decay function	β	0.113	0.117	0.114	0.106	0.108	0.107	0.103	0.102	0.101	0.102
	*R* ^2^	0.946	0.947	0.951	0.962	0.958	0.962	0.966	0.968	0.966	0.966

a β = decay parameter; *R* ^2 ^= the decay function’s fitness to data.

Year		2005	2006	2007	2008	2009	2010	2011	2012	2013	2014
Decay function	β	0.113	0.117	0.114	0.106	0.108	0.107	0.103	0.102	0.101	0.102
	*R* ^2^	0.946	0.947	0.951	0.962	0.958	0.962	0.966	0.968	0.966	0.966

## Discussion

Analysis of ACS data indicates that the percentage of travel to work by walking increased slightly from 2005 through 2014 and that the people who walked to work walked longer. Although the increases were small, they are positive and encouraging signs in light of the decrease in the percentage of active travel to work over the past several decades in the United States.

The choice of travel mode is a complicated function and is influenced by multiple factors. In the United States, the increase of gasoline prices in the past decade may partly explain the increase of walking trips. Additionally, people who lived longer distances from their workplace may have been more likely to switch to working at home because of recent advancements in information technology. The pattern observed from ACS data is an aggregation of national-level data, and the changes of people and context are unlikely to be even across various population groups and regions.

One limitation of this study is that it is based on a single purpose. Walking to work, although an important component of all walking trips overall, may be less susceptible to change than walking for other purposes. Another limitation is that ACS data do not provide the trip distance, and relying on duration ignores the variation of walking speed. Finally, data were self-reported and are therefore susceptible to bias and memory.

This study, although simple, provides sufficient evidence to prompt research on data collection and analysis related to walking to work, and these study results could serve as a catalyst for more aggressive policy intervention.
